# *N*-[(*E*)-2,4-Dichloro­benzyl­idene]-4-methyl­aniline

**DOI:** 10.1107/S1600536810036640

**Published:** 2010-09-18

**Authors:** M. Nawaz Tahir, Hazoor Ahmad Shad, Muhammad Ilyas Tariq, Riaz H. Tariq

**Affiliations:** a Department of Physics, University of Sargodha, Sargodha, Pakistan; b Department of Chemistry, Govt. M. D. College, Toba Tek Singh, Punjab, Pakistan; c Department of Chemistry, University of Sargodha, Sargodha, Pakistan; d Institute of Chemical and Pharmaceutical Sciences, The University of Faisalabad, Faisalabad, Pakistan

## Abstract

In the title compound, C_14_H_11_Cl_2_N, the dihedral angle between the 4-methyl­anilinic and 2,4-dichloro­benzaldehyde moieties is 7.37 (8)°. In the crystal, C—H⋯π inter­actions between the terminal methyl group and a symmetry-related ring of the anilinic group help to establish the packing.

## Related literature

For background to our project to synthesize various Schiff bases of 2,4-dichloro­benzaldehyde as possible ligands for complexing metals, see: Hayat *et al.* (2010 ▶). For related structures, see: Hayat *et al.* (2010 ▶); Bernstein (1972 ▶). For graph-set notation, see: Bernstein *et al.* (1995 ▶).
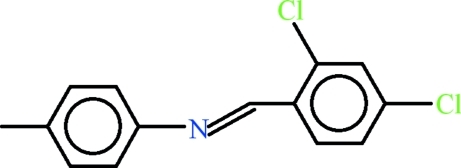


## Experimental

### 

#### Crystal data

C_14_H_11_Cl_2_N
*M* *_r_* = 264.14
Monoclinic, 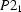

*a* = 10.1069 (3) Å
*b* = 4.7469 (2) Å
*c* = 12.9922 (4) Å
β = 95.668 (2)°
*V* = 620.27 (4) Å^3^
*Z* = 2
Mo *K*α radiation
μ = 0.50 mm^−1^
*T* = 296 K
0.32 × 0.20 × 0.18 mm

#### Data collection

Bruker Kappa APEXII CCD diffractometer
Absorption correction: multi-scan (*SADABS*; Bruker, 2005 ▶) *T*_min_ = 0.886, *T*_max_ = 0.916
5221 measured reflections
2082 independent reflections
1937 reflections with *I* > 2σ(*I*)
*R*_int_ = 0.022

#### Refinement

*R*[*F* ^2^ > 2σ(*F* ^2^)] = 0.030
*wR*(*F* ^2^) = 0.079
*S* = 1.06
2082 reflections
155 parameters
1 restraint
H-atom parameters constrained
Δρ_max_ = 0.16 e Å^−3^
Δρ_min_ = −0.15 e Å^−3^
Absolute structure: Flack (1983 ▶), 807 Friedel pairs
Flack parameter: 0.10 (7)

### 

Data collection: *APEX2* (Bruker, 2009 ▶); cell refinement: *SAINT* (Bruker, 2009 ▶); data reduction: *SAINT*; program(s) used to solve structure: *SHELXS97* (Sheldrick, 2008 ▶); program(s) used to refine structure: *SHELXL97* (Sheldrick, 2008 ▶); molecular graphics: *ORTEP-3 for Windows* (Farrugia, 1997 ▶); software used to prepare material for publication: *WinGX* (Farrugia, 1999 ▶) and *PLATON* (Spek, 2009 ▶).

## Supplementary Material

Crystal structure: contains datablocks global, I. DOI: 10.1107/S1600536810036640/dn2602sup1.cif

Structure factors: contains datablocks I. DOI: 10.1107/S1600536810036640/dn2602Isup2.hkl

Additional supplementary materials:  crystallographic information; 3D view; checkCIF report

## Figures and Tables

**Table 1:** Hydrogen-bond geometry (Å, °) *Cg*1 is the centroid of the C1–C6 ring.

*D*—H⋯*A*	*D*—H	H⋯*A*	*D*⋯*A*	*D*—H⋯*A*
C7—H7*C*⋯*Cg*1^i^	0.96	2.71	3.565 (2)	148

Symmetry code: (i) 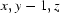.

**Fig. 1.**
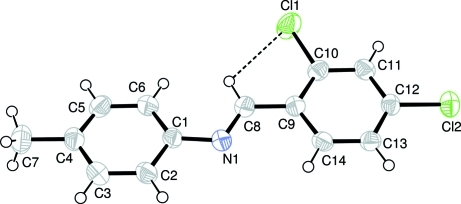
View of the title compound with the atom numbering scheme. The thermal ellipsoids are drawn at the 50% probability level. H-atoms are shown as small circles of arbitrary radii. The dotted line represents the intramolecular H-bonding.

## supplementary crystallographic information

### Comment

As a part of our on going project related to synthesize various Schiff bases of 2,4-dichlorobenzaldehyde as possible ligands for complexing metals (Hayat *et al.*, 2010), we report here the title compound.

In the title compound, the 4-methylanilinic group A (C1—C7/N1) and 2,4-dichlorobenzaldehyde moiety B (C8—C14/CL1/CL2) are planar with r. m. s. deviation of 0.0114 and 0.0209 Å, respectively. The dihedral angle between A/B is 7.37 (8)°. The title compound essentially consists of monomers (Fig. 1). There exist weak intramolecular C—H···Cl hydrogen bonds (Table 1, Fig. 1) forming an S(5) ring motif (Bernstein *et al.*, 1995). There also exists a C—H···π interaction (Table 1) which helps in consolidating the crystal packing. Bond distances and bond angles agree with related compounds already published as the 4-chloro-*N*-[(*E*)-2,4-dichlorobenzylidene]aniline (Hayat *et al.*, 2010) and the *N*-(2,4-dichlorobenzylidene)aniline (Bernstein, 1972).

### Experimental

Equimolar quantities of 4-methylaniline and 2,4-dichlorobanzaldehyde were refluxed in methanol for 30 min resulting in yellow solution. The solution was kept at room temperature which affoarded light yellow needles after 72 h.

### Refinement

The H-atoms were positioned geometrically (C–H = 0.93–0.96 Å) and were included in the refinement in the riding model approximation, with *U*_iso_(H) = *xU*_eq_(C), where *x* = 1.5 for methyl H-atoms and *x* = 1.2 for aryl H-atoms.

### Crystal data

**Table:**

C_14_H_11_Cl_2_N	*F*(000) = 272
*M**_r_* = 264.14	*D*_x_ = 1.414 Mg m^−^^3^
Monoclinic, *P*2_1_	Mo *K*α radiation, λ = 0.71073 Å
Hall symbol: P 2yb	Cell parameters from 1937 reflections
*a* = 10.1069 (3) Å	θ = 1.6–25.2°
*b* = 4.7469 (2) Å	µ = 0.50 mm^−^^1^
*c* = 12.9922 (4) Å	*T* = 296 K
β = 95.668 (2)°	Needles, colorless
*V* = 620.27 (4) Å^3^	0.32 × 0.20 × 0.18 mm
*Z* = 2	

### Data collection

**Table:**

Bruker Kappa APEXII CCD diffractometer	2082 independent reflections
Radiation source: fine-focus sealed tube	1937 reflections with *I* > 2σ(*I*)
graphite	*R*_int_ = 0.022
Detector resolution: 8.20 pixels mm^-1^	θ_max_ = 25.2°, θ_min_ = 1.6°
ω scans	*h* = −12→12
Absorption correction: multi-scan (*SADABS*; Bruker, 2005)	*k* = −5→4
*T*_min_ = 0.886, *T*_max_ = 0.916	*l* = −15→15
5221 measured reflections	

### Refinement

**Table:**

Refinement on *F*^2^	Secondary atom site location: difference Fourier map
Least-squares matrix: full	Hydrogen site location: inferred from neighbouring sites
*R*[*F*^2^ > 2σ(*F*^2^)] = 0.030	H-atom parameters constrained
*wR*(*F*^2^) = 0.079	*w* = 1/[σ^2^(*F*_o_^2^) + (0.0434*P*)^2^ + 0.0661*P*] where *P* = (*F*_o_^2^ + 2*F*_c_^2^)/3
*S* = 1.06	(Δ/σ)_max_ < 0.001
2082 reflections	Δρ_max_ = 0.16 e Å^−^^3^
155 parameters	Δρ_min_ = −0.14 e Å^−^^3^
1 restraint	Absolute structure: Flack (1983), 807 Friedel pairs
Primary atom site location: structure-invariant direct methods	Flack parameter: 0.10 (7)

### Special details

**Table:**

Geometry. Bond distances, angles *etc*. have been calculated using the rounded fractional coordinates. All su's are estimated from the variances of the (full) variance-covariance matrix. The cell e.s.d.'s are taken into account in the estimation of distances, angles and torsion angles
Refinement. Refinement of *F*^2^ against ALL reflections. The weighted *R*-factor *wR* and goodness of fit *S* are based on *F*^2^, conventional *R*-factors *R* are based on *F*, with *F* set to zero for negative *F*^2^. The threshold expression of *F*^2^ > σ(*F*^2^) is used only for calculating *R*-factors(gt) *etc*. and is not relevant to the choice of reflections for refinement. *R*-factors based on *F*^2^ are statistically about twice as large as those based on *F*, and *R*- factors based on ALL data will be even larger.

### Fractional atomic coordinates and isotropic or equivalent isotropic displacement parameters (Å^2^)

**Table:**

	*x*	*y*	*z*	*U*_iso_*/*U*_eq_	
Cl1	−0.16693 (6)	0.51040 (18)	0.89821 (4)	0.0670 (3)	
Cl2	−0.39799 (5)	1.07125 (13)	0.57256 (4)	0.0492 (2)	
N1	0.08154 (17)	0.0949 (5)	0.69281 (13)	0.0393 (6)	
C1	0.1724 (2)	−0.1021 (5)	0.74152 (15)	0.0359 (7)	
C2	0.2670 (2)	−0.2162 (5)	0.68218 (17)	0.0436 (8)	
C3	0.3592 (2)	−0.4089 (6)	0.72278 (18)	0.0488 (8)	
C4	0.3619 (2)	−0.4993 (5)	0.82462 (16)	0.0422 (8)	
C5	0.2663 (2)	−0.3948 (6)	0.88216 (16)	0.0478 (8)	
C6	0.1728 (2)	−0.1997 (6)	0.84251 (16)	0.0450 (8)	
C7	0.4635 (3)	−0.7104 (6)	0.8693 (2)	0.0601 (10)	
C8	0.0090 (2)	0.2361 (5)	0.74719 (16)	0.0396 (7)	
C9	−0.08867 (19)	0.4437 (5)	0.70395 (15)	0.0354 (6)	
C10	−0.17493 (19)	0.5787 (6)	0.76574 (14)	0.0394 (6)	
C11	−0.2697 (2)	0.7695 (5)	0.72713 (16)	0.0408 (7)	
C12	−0.2778 (2)	0.8341 (5)	0.62295 (16)	0.0359 (7)	
C13	−0.1920 (2)	0.7130 (5)	0.55900 (15)	0.0376 (7)	
C14	−0.10045 (19)	0.5191 (5)	0.59983 (14)	0.0378 (7)	
H2	0.26757	−0.16057	0.61359	0.0523*	
H3	0.42111	−0.48034	0.68121	0.0586*	
H5	0.26408	−0.45686	0.94987	0.0574*	
H6	0.10958	−0.13313	0.88390	0.0540*	
H7A	0.45868	−0.72613	0.94246	0.0901*	
H7B	0.55083	−0.64872	0.85642	0.0901*	
H7C	0.44563	−0.89053	0.83730	0.0901*	
H8	0.01785	0.20723	0.81833	0.0475*	
H11	−0.32689	0.85279	0.77004	0.0490*	
H13	−0.19621	0.76212	0.48946	0.0452*	
H14	−0.04426	0.43496	0.55632	0.0453*	

### Atomic displacement parameters (Å^2^)

**Table:**

	*U*^11^	*U*^22^	*U*^33^	*U*^12^	*U*^13^	*U*^23^
Cl1	0.0692 (4)	0.0965 (7)	0.0371 (3)	0.0234 (4)	0.0140 (3)	0.0138 (3)
Cl2	0.0445 (3)	0.0449 (4)	0.0571 (3)	0.0089 (3)	−0.0002 (2)	0.0058 (3)
N1	0.0421 (9)	0.0365 (12)	0.0392 (8)	0.0048 (9)	0.0034 (7)	0.0005 (9)
C1	0.0374 (11)	0.0300 (13)	0.0394 (11)	0.0005 (9)	−0.0007 (8)	−0.0031 (9)
C2	0.0509 (13)	0.0423 (15)	0.0388 (11)	0.0090 (12)	0.0108 (10)	0.0015 (10)
C3	0.0487 (12)	0.0460 (15)	0.0524 (12)	0.0099 (14)	0.0083 (10)	−0.0053 (13)
C4	0.0413 (12)	0.0301 (15)	0.0529 (12)	−0.0007 (10)	−0.0063 (9)	−0.0033 (11)
C5	0.0565 (13)	0.0481 (17)	0.0373 (10)	0.0021 (13)	−0.0029 (9)	0.0031 (11)
C6	0.0497 (13)	0.0473 (15)	0.0384 (11)	0.0087 (12)	0.0062 (9)	−0.0025 (10)
C7	0.0529 (15)	0.0483 (17)	0.0748 (18)	0.0085 (13)	−0.0145 (12)	0.0017 (14)
C8	0.0419 (12)	0.0385 (14)	0.0380 (10)	0.0012 (10)	0.0026 (9)	0.0012 (10)
C9	0.0330 (10)	0.0335 (12)	0.0394 (10)	−0.0025 (10)	0.0016 (8)	−0.0012 (10)
C10	0.0415 (10)	0.0443 (14)	0.0328 (9)	−0.0009 (12)	0.0057 (8)	0.0030 (11)
C11	0.0399 (11)	0.0410 (15)	0.0424 (11)	0.0039 (10)	0.0089 (9)	−0.0022 (10)
C12	0.0352 (11)	0.0290 (12)	0.0426 (11)	−0.0022 (9)	−0.0004 (8)	0.0011 (9)
C13	0.0419 (12)	0.0360 (13)	0.0348 (10)	−0.0011 (10)	0.0027 (9)	−0.0014 (10)
C14	0.0370 (10)	0.0388 (15)	0.0378 (10)	0.0020 (10)	0.0055 (8)	−0.0060 (11)

### Geometric parameters (Å, °)

**Table:**

Cl1—C10	1.7454 (19)	C11—C12	1.382 (3)
Cl2—C12	1.736 (2)	C12—C13	1.384 (3)
N1—C1	1.415 (3)	C13—C14	1.374 (3)
N1—C8	1.260 (3)	C2—H2	0.9300
C1—C2	1.396 (3)	C3—H3	0.9300
C1—C6	1.391 (3)	C5—H5	0.9300
C2—C3	1.373 (3)	C6—H6	0.9300
C3—C4	1.389 (3)	C7—H7A	0.9600
C4—C5	1.372 (3)	C7—H7B	0.9600
C4—C7	1.509 (4)	C7—H7C	0.9600
C5—C6	1.386 (3)	C8—H8	0.9300
C8—C9	1.467 (3)	C11—H11	0.9300
C9—C10	1.398 (3)	C13—H13	0.9300
C9—C14	1.393 (3)	C14—H14	0.9300
C10—C11	1.376 (3)		
			
Cl1···C6^i^	3.519 (2)	C1···H7C^viii^	3.0800
Cl2···Cl2^ii^	3.5598 (8)	C2···H7C^viii^	3.0000
Cl2···Cl2^iii^	3.5598 (8)	C3···H7C^viii^	2.9600
Cl1···H7B^iv^	2.9500	C4···H7C^viii^	3.0100
Cl1···H6^i^	2.9100	C5···H7C^viii^	3.0900
Cl1···H8	2.6500	C6···H8	2.4900
Cl2···H2^v^	3.1400	C8···H6	2.6300
N1···C14^vi^	3.446 (3)	C13···H2^x^	2.9000
N1···H14	2.6300	H2···Cl2^xi^	3.1400
N1···H13^vii^	2.8500	H2···C13^vii^	2.9000
C1···C4^viii^	3.552 (3)	H2···H13^vii^	2.4800
C1···C8^vi^	3.553 (3)	H5···H7A	2.3600
C1···C9^vi^	3.405 (3)	H6···C8	2.6300
C4···C1^vi^	3.552 (3)	H6···H8	2.0100
C5···C8^vi^	3.465 (3)	H6···Cl1^ix^	2.9100
C6···C9^vi^	3.486 (3)	H7A···H5	2.3600
C6···C8^vi^	3.323 (3)	H7B···Cl1^xii^	2.9500
C6···Cl1^ix^	3.519 (2)	H7C···C1^vi^	3.0800
C8···C6^viii^	3.323 (3)	H7C···C2^vi^	3.0000
C8···C1^viii^	3.553 (3)	H7C···C3^vi^	2.9600
C8···C5^viii^	3.465 (3)	H7C···C4^vi^	3.0100
C8···C11^vi^	3.573 (3)	H7C···C5^vi^	3.0900
C9···C6^viii^	3.486 (3)	H8···Cl1	2.6500
C9···C1^viii^	3.405 (3)	H8···C6	2.4900
C9···C12^vi^	3.569 (3)	H8···H6	2.0100
C11···C8^viii^	3.573 (3)	H13···N1^x^	2.8500
C12···C9^viii^	3.569 (3)	H13···H2^x^	2.4800
C14···N1^viii^	3.446 (3)	H14···N1	2.6300
			
C1—N1—C8	119.23 (18)	C9—C14—C13	122.46 (19)
N1—C1—C2	117.33 (18)	C1—C2—H2	119.00
N1—C1—C6	125.67 (19)	C3—C2—H2	119.00
C2—C1—C6	117.0 (2)	C2—C3—H3	119.00
C1—C2—C3	121.5 (2)	C4—C3—H3	119.00
C2—C3—C4	121.4 (2)	C4—C5—H5	119.00
C3—C4—C5	117.3 (2)	C6—C5—H5	119.00
C3—C4—C7	121.4 (2)	C1—C6—H6	120.00
C5—C4—C7	121.3 (2)	C5—C6—H6	120.00
C4—C5—C6	122.0 (2)	C4—C7—H7A	109.00
C1—C6—C5	120.8 (2)	C4—C7—H7B	109.00
N1—C8—C9	123.32 (19)	C4—C7—H7C	109.00
C8—C9—C10	121.49 (18)	H7A—C7—H7B	110.00
C8—C9—C14	122.30 (19)	H7A—C7—H7C	110.00
C10—C9—C14	116.21 (19)	H7B—C7—H7C	109.00
Cl1—C10—C9	120.69 (17)	N1—C8—H8	118.00
Cl1—C10—C11	116.39 (16)	C9—C8—H8	118.00
C9—C10—C11	122.92 (18)	C10—C11—H11	121.00
C10—C11—C12	118.33 (19)	C12—C11—H11	121.00
Cl2—C12—C11	118.99 (16)	C12—C13—H13	121.00
Cl2—C12—C13	119.97 (16)	C14—C13—H13	120.00
C11—C12—C13	121.0 (2)	C9—C14—H14	119.00
C12—C13—C14	118.98 (18)	C13—C14—H14	119.00
			
C8—N1—C1—C2	−168.7 (2)	N1—C8—C9—C14	−5.9 (4)
C8—N1—C1—C6	13.7 (4)	C8—C9—C10—Cl1	1.5 (3)
C1—N1—C8—C9	−179.6 (2)	C8—C9—C10—C11	−178.5 (2)
N1—C1—C2—C3	−180.0 (2)	C14—C9—C10—Cl1	−178.14 (17)
C6—C1—C2—C3	−2.2 (3)	C14—C9—C10—C11	2.0 (3)
N1—C1—C6—C5	179.6 (2)	C8—C9—C14—C13	179.9 (2)
C2—C1—C6—C5	2.0 (3)	C10—C9—C14—C13	−0.6 (3)
C1—C2—C3—C4	0.2 (4)	Cl1—C10—C11—C12	178.75 (18)
C2—C3—C4—C5	1.9 (4)	C9—C10—C11—C12	−1.3 (4)
C2—C3—C4—C7	−179.8 (2)	C10—C11—C12—Cl2	179.26 (18)
C3—C4—C5—C6	−2.1 (4)	C10—C11—C12—C13	−0.7 (3)
C7—C4—C5—C6	179.6 (2)	Cl2—C12—C13—C14	−177.94 (17)
C4—C5—C6—C1	0.2 (4)	C11—C12—C13—C14	2.1 (3)
N1—C8—C9—C10	174.6 (2)	C12—C13—C14—C9	−1.4 (3)

Symmetry codes: (i) −*x*, *y*+1/2, −*z*+2; (ii) −*x*−1, *y*−1/2, −*z*+1; (iii) −*x*−1, *y*+1/2, −*z*+1; (iv) *x*−1, *y*+1, *z*; (v) −*x*, *y*+3/2, −*z*+1; (vi) *x*, *y*−1, *z*; (vii) −*x*, *y*−1/2, −*z*+1; (viii) *x*, *y*+1, *z*; (ix) −*x*, *y*−1/2, −*z*+2; (x) −*x*, *y*+1/2, −*z*+1; (xi) −*x*, *y*−3/2, −*z*+1; (xii) *x*+1, *y*−1, *z*.

### Hydrogen-bond geometry (Å, °)

**Table:**

Cg1 is the centroid of the C1–C6 phenyl ring.

**Table:**

*D*—H···*A*	*D*—H	H···*A*	*D*···*A*	*D*—H···*A*
C8—H8···Cl1	0.93	2.65	3.065 (2)	108
C7—H7C···Cg1^vi^	0.96	2.71	3.565 (2)	148

Symmetry codes: (vi) *x*, *y*−1, *z*.
